# The Effect of Sandblasting on Bond Strength of Soft Liners to Denture Base Resins: A Systematic Review and Meta-Analysis of In Vitro Studies

**DOI:** 10.1155/2021/5674155

**Published:** 2021-12-22

**Authors:** Fahimeh Hamedirad, Marzieh Alikhasi, Mahya Hasanzade

**Affiliations:** ^1^Dental Materials Research Center, Health Research Institute, Babol University of Medical Sciences, Babol, Iran; ^2^Dental Research Center and Dental Implant Research Center, Dentistry Research Institute, Department of Prosthodontics, School of Dentistry, Tehran University of Medical Sciences, Tehran, Iran; ^3^Dental Research Center, Dentistry Research Institute, Department of Prosthodontics, School of Dentistry, Tehran University of Medical Sciences, Tehran, Iran

## Abstract

**Objectives:**

This study aimed to evaluate the effect of sandblasting on the bond strength of denture base resin to soft liners.

**Materials and Methods:**

This report follows the Preferred Reporting Items for Systematic Reviews and Meta-Analyses (PRISMA) statement. PubMed, Embase, Cochrane, Scopus, and OpenGrey databases were searched for in vitro studies that compared sandblasting with no treatment in terms of the tensile, shear, and peel bond strength of resilient lining materials (acrylic-based or silicone-based) to polymethyl methacrylate denture base resin. Based on the outcome, the analysis was carried out in three groups of tensile, shear, and peel bond strength. Subgroup analysis was done for the effect of size of particles on sandblasting, blasting pressure, and type of soft liner whenever possible. Heterogeneity was evaluated among the studies, and meta-analysis was performed with random effect models (*p* < .05).

**Results:**

After screening, 16 articles met the inclusion criteria for meta-analyses. No treatment showed significantly higher tensile (*p* < 0.001) or peel (*p*=0.04) bond strength, although shear bond strength of sandblasted resin was significantly better (*p*=0.008). Results of subgroup analyses of particle size favored the control group in 50 *µ* Al_2_O_3_ particle size (*p* < 0.001). In analyses of blasting pressure, the control group had significantly better tensile bond strength than specimens with blasting pressure ≤1 bar (*p* < 0.001) while specimens with blasting pressure beyond 1 bar showed significantly more tensile strength than control group (*p*=0.03). In silicon-based liners, groups without any surface treatment had significantly higher tensile bond strength (*p* < 0.001).

**Conclusion:**

According to the in vitro studies, sandblasting would not lead to significant increase in bond strength of soft liner to the denture base resin.

## 1. Introduction

Prolonged use of dentures is common among elderly patients. It could cause denture soreness and serve bone resorption [1]. Resilient lining materials are used to distribute the pressure equally and prevent localization of force by a cushion effect under the denture bases [2–11]. Relining materials offer dentists a quick, convenient, and short time solution for patient problems. Indications of resilient lining materials are seen in patients with exostosis due to uneven bone resorption, tender soft tissues, bony undercuts, immediate dentures, treatment dentures after implantation or healing period, presence of parafunctional habits, xerostomia, ill fitted dentures, wearing facial prostheses, and demand for better rhythm of chewing strokes. They also compensate for the volumetric shrinkage of acrylic resin [6, 12–15]. These materials can be provisional or permanent, and auto- or heat-cure-polymerized [16–18]. Five types of soft liners exist according to their chemical structures, namely, plasticized acrylic resins (chemical or heat-polymerized), vinyl resins, polyurethane, polyphosphazene, and silicone rubbers (heat-cured or room-temperature-vulcanized) [8]. All types of resilient liner materials have some drawbacks such as insufficient color stability, losing resiliency over time, poor abrasion resistance, presence of surface defects and porosity, water uptake, microbial gathering, bond failures to denture base resin, unsatisfied taste over the time, mephitis, difficulty in cleaning, and premature hardening due to plasticizers solubilization [12, 19–24].

Two-layer dentures can only be successful when there is strong adhesion between different layers of materials [25] Tensile bond strength with a minimum of 0.44 MPa (4.5 kg/cm^2^) between acrylic resin and liner is needed to be acceptable for clinical usage [26–28]. To overcome the weak bond strength between denture base resin (DBR) and liners, sandblasting with alumina, laser application, chemical cauterization or primers, acrylic drills, or mesh textured glass fibers have been used by researchers [2, 6, 9, 11, 29–33]. The investigators tried to roughen DBR surface with airborne particles before adding the liners to improve the bond strength [26, 31, 34–37]. Controversy exists regarding efficiency of sandblasting in improving bond strength. While some investigations have shown improved bond strength, [30, 32] others have reported that mechanical surface treatment of DBR decreases the adhesion bond strength [21, 25, 29, 31]. Meanwhile, existing reviews evaluated sandblasting without considering the role of sandblasting parameters in the final outcome. The aim of this systematic review was to analyze the effect of sandblasting on bond strength of resilient lining materials applied to DBR considering size of particles in sandblasting, blasting pressure, and type of soft liner.

## 2. Materials and Methods

This systematic review was reported according to the PRISMA (Preferred Reporting Items for Systematic Reviews and Meta-Analyses) statement [38, 39]. The study design focuses on answering the following question, according to PICO strategy: “what is the effect of sandblasting on bond strength of resilient liners to polymethyl methacrylate (PMMA)?” In this process, the population was denture base resins that were bonded to soft liners; the intervention was sandblasting the substrate; the comparison was made with groups without any surface treatment; and the outcomes were tensile bond strength, shear bond strength, or peel bond strength between acrylic denture base and soft liner. The secondary aim of the study was to answer two further questions: “What is the effect of Al_2_O_3_ particle size and blasting pressure on the bond strength between denture base resin and resilient liner materials?” “How could the type of soft liners affect the bond of sandblasted groups and control groups?” The review question, aims of the study, suitability criteria, search strategy, and data analysis were specified in the beginning with clarity and were included in the study content. A systematic literature search was done in the PubMed, Embase, Cochrane, Scopus, and OpenGrey (https://www.opengrey.eu) databases until January 2020 (Table 1). Furthermore, the reference and citations' lists of the selected articles were reviewed for selecting potential inclusions.

Eligible studies were experimental, in vitro, and laboratory studies which evaluated the bond strength of resilient lining materials (acrylic-based or silicone-based) to polymethyl methacrylate (PMMA) denture base resin and compared no treatment (control) with sandblasting surface pretreatment (experimental) in the same study. In addition, the study should report the mean and standard deviation (SD) of tensile, shear, or peel bond strength. Studies that evaluated other materials for denture base except PPMA, critiques, case reports, systematic reviews, and expert opinion papers were excluded. 38 studies that did not provide essential data after contacting the authors via e-mail were also excluded. Moreover, included studies had to be published in English.

Title, abstract, and full text selection were carried out by two authors (F. H. and M. A.) independently. Finally selected full text studies, based on inclusion and exclusion criteria, were those with proper control group having no surface treatment and with experimental group in which no further treatment, such as applying adhesive, was performed after sandblasting. Disagreements on selection process were resolved by a third investigator (M. H.), and finally consensus was reached through discussion. Two investigators extracted study content and data independently using a standard form prepared in software (Office Excel 2013 software, Microsoft Corporation, Redmond, WA, USA). The following data were extracted: sample size, name of acrylic, liner material, particle size of sandblasting, pressure of sandblasting, time of sandblasting, distance from sandblasting tip to specimen, storage condition of specimen before testing, thermocycling, mean and SD of experimental and control group, and failure mode. Any disagreements between investigators were resolved by discussion. In studies where enough information was not provided, the authors were contacted via e-mail.

Two authors (F. H. and M. H.) evaluated the methodological quality of each enrolled study independently bases on reported tools of previous systematic reviews of in vitro studies. [40, 41] Therefore, the following parameters were checked for risk of bias evaluation: specimen randomization, single operator protocol implementation, blinding of the testing machine operator, presence of a control group, standardization of sample preparation, failure mode evaluation, use of materials according to the manufacturer's recommendation, description of sample size calculation, and discarded unacceptable samples. If the article reported the parameter, it received “yes” for that parameter. If information is not provided or the article does not follow the parameters, it received “not mentioned” or “no,” respectively. Articles with one to three reported items were considered as high risk of bias, four to five as medium risk of bias, and six to nine as low risk of bias.

For meta-analysis, the outcomes were categorized into three groups of tensile, shear, and peel bond strength. Sandblasted and control groups were analyzed in each category both globally and by subgroups. The effect of size of particles on sandblasting, blasting pressure, and type of soft liner was analyzed in subgroups in categories with sufficient data. Studies with several independent experimental and control groups were assumed as independent comparisons in meta-analysis. For studies with multiple correlated comparisons (control group in common), groups were combined with specific formula for mean and SD to create a single pairwise comparison in order to overcome a unit-of-analysis error.

Meta-analysis was based on inverse-variance method. As MPa was accepted unit for reporting bond strength values, values of different units were converted to MPa. Bond strength was the continuous outcome evaluated for mean difference (MD) and the corresponding confidence interval. A *p* value ≤0.05 was considered statistically significant in *Z* test. Heterogeneity among studies was calculated using *I*^2^ and chi^2^ tests. All analyses were done using random effect model in Review Manager software (version 5.1, Cochrane Collaboration, Copenhagen, Denmark).

## 3. Results

**Figure 1:**
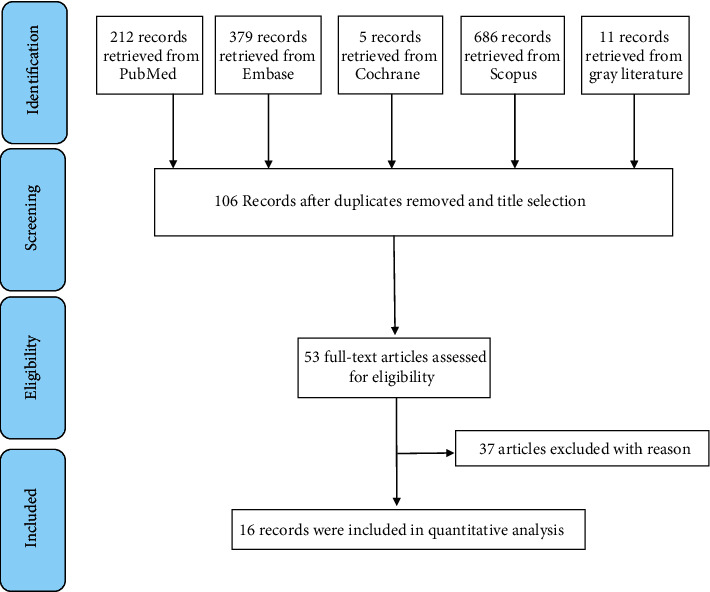
Flow diagram of screening the title, abstract, and full text.

The process of screening the articles is summarized in Figure 1 according to PRISMA statement. 106 articles were identified from databases after reading the titles, of these 53 were eligible for full text evaluation. Finally, 37 studies were excluded for the reasons presented in Table 2, and 16 articles were enrolled for meta-analysis. Study characteristics and descriptive evaluation of studies are presented in Table 3.

**Figure 2:**
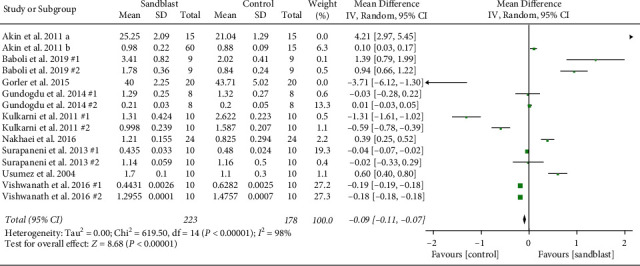
Forest plot for global analysis of tensile bond strength.

**Figure 3:**
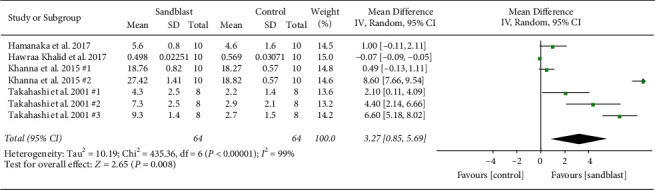
Forest plot for global analysis of shear bond strength.

**Figure 4:**
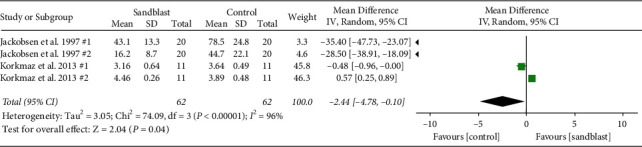
Forest plot for global analysis of peel bond strength.

Overall, eight meta-analyses were done, three global and five subgroup analyses. At the first global analysis for tensile bond strength, 15 pair comparisons from ten studies were analyzed. Results showed that control group had significantly higher bond strength in comparison to blasting group (*p* < 0.001) (Figure 2). At the second analysis, global analysis of shear bond strength was carried out with seven pairs from four articles. In this analysis, statistical difference was found (*p*=0.008) favoring the group subjected to sandblasting (Figure 3). The third global analysis of peel strength included four pairs from two articles. The results showed significant difference between experimental and control group with higher bond strength in control group (*p*=0.04) (Figure 4). In all analyses *I*^2^ was beyond 95%, indicating high heterogeneity.

**Figure 5:**
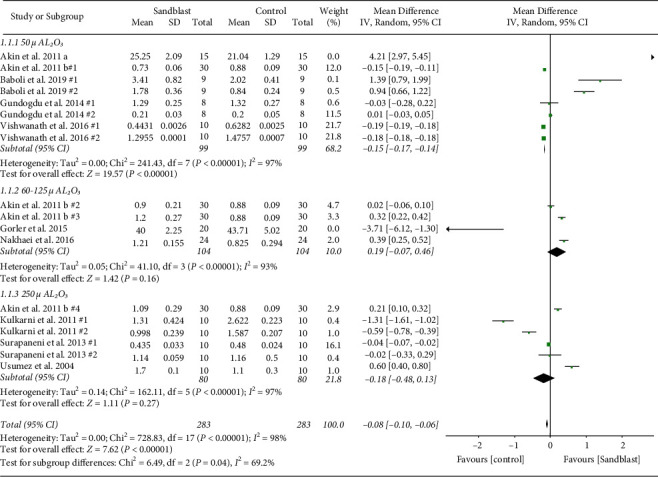
Forest plot for subgroup analysis of particle size for tensile bond strength.

**Figure 6:**
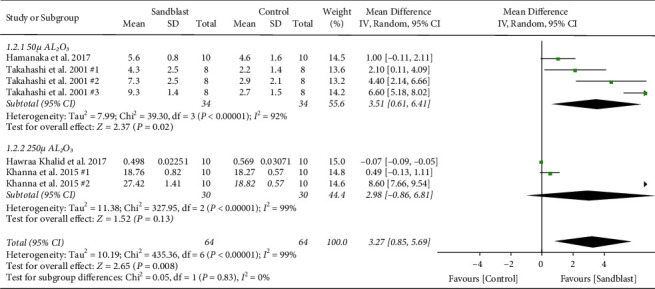
Forest plot for subgroup analysis of particle size for shear bond strength.

**Figure 7:**
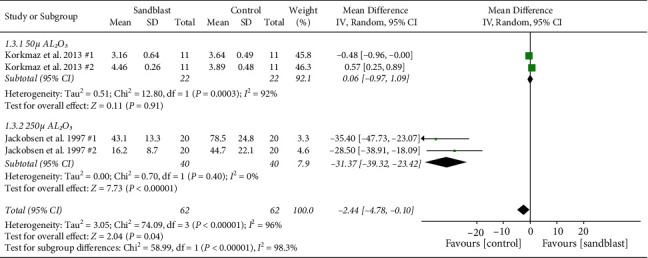
Forest plot for subgroup analysis of particle size for peel bond strength.

First subgroup analysis was particle size of sandblasting. The studies were categorized into three groups with strata of small size particle (50 *µ* Al_2_O_3_), medium size (50 *µ* Al_2_O_3_ < particle size< 250 *µ* Al_2_O_3_), and large size (particle size ≥250 *µ* Al_2_O_3_). The MD of subgroups in tensile bond strength is presented in Figure 5. The results favored control group in 5000a0*µ* Al_2_O_3_ particle size (*p* < 0.001). However, as the particle size went beyond 50 *µ*, the effect was nonsignificant. In particle size subgroup analysis of shear bond strength, sandblasting with 50 *µ* Al_2_O_3_ resulted in significantly higher shear bond strength (*p*=0.02). Groups which were sandblasted with 250 *µ* Al_2_O_3_ had no significant difference with no treatment specimens (Figure 6). Evaluating the effect of particle size in peel strength resulted in two groups from one study for each of 50 and 250 *µ* Al_2_O_3_ categories. Korkmaz et al. evaluated the peel strength between control and 50 *µ* Al_2_O_3_ sandblasting and showed no significant difference. [32] However, when 250 *µ* Al_2_O_3_ was used for treating the PMMA in Jacobsen's study, the results were significant, favoring sandblasted groups (*p* < 0.001) (Figure 7) [31].

**Figure 8:**
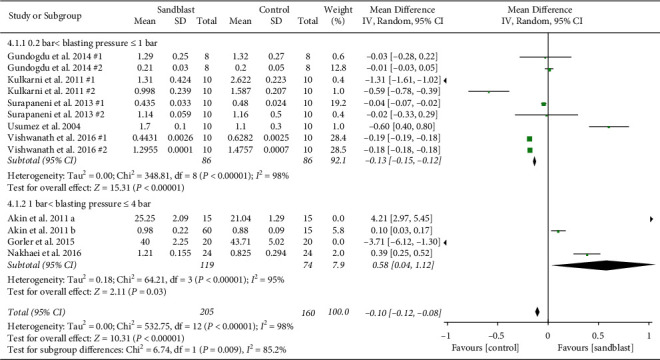
Forest plot for subgroup analysis of blasting pressure for tensile bond strength.

The second subgroup analysis was performed to investigate the effect of blasting pressure. Pair comparison groups were categorized into two strata based on blasting pressure (0.2 bar ≤ blasting pressure ≤1 bar; 1 bar < blasting pressure ≤ 4 bar). Meta-analysis showed higher tensile bond strength for control group when the blasting pressure was ≤1 bar (*p* < 0.001). By increasing the blasting pressure beyond 1 bar, sandblasting became significantly more effective than control group (*p*=0.03) (Figure 8).

**Figure 9:**
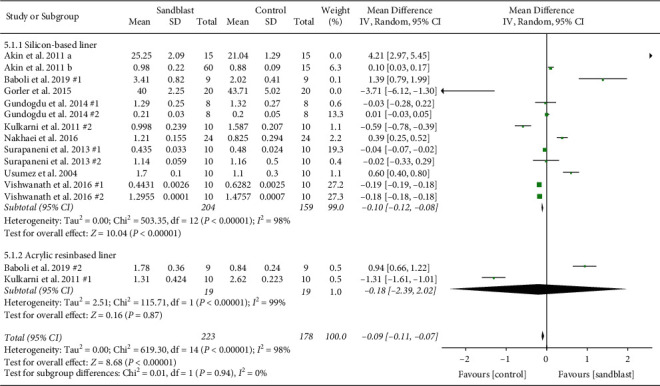
Forest plot for subgroup analysis of type of soft liner for tensile bond strength.

The effect of type of soft liner was investigated with strata of silicon-based liner and acrylic resin-based liner. As study groups were not sufficient in shear and peel bond strength categories, this subgroup analysis was only conducted for tensile bond strength. The results showed that groups without any surface treatment had significantly higher tensile bond strength when silicon-based liner was used (*p* < 0.001). Meanwhile, the two studies that used acrylic resin-based liners showed no significant difference between control and sandblasting groups (Figure 9).

Results of quality assessment showed that six studies had medium risk of bias and ten studies had low risk of bias (Table 4). The most not reported items were “single operator protocol implementation” and “blinding of the testing machine operator.”

## 4. Discussion

Different methods have been introduced to improve bond of denture base resins to soft liners. The influence of these methods has been evaluated in two systematic reviews. [84, 85] The enhancement mechanisms can be divided into three general categories: first, increasing the available surface area for bonding by increasing surface roughness; second, improving the chemical behavior of substrate to improve wettability; and finally establishing hydrogen bond between acrylic group of PMMA and adhesive primers. Treating the surface by laser, sandblasting, and chemical solvent influences the bond strength through increasing surface roughness. The surface of material that is candidate for bonding can be sandblasted by spraying a stream of Al_2_O_3_ particles under high pressure. [86] Global results from two systematic reviews showed that airborne particle abrasion decreases the bond strength between denture base resin and soft liners. [84, 85] However, this result contradicts a number of studies that showed higher bond strength after sandblasting. [29, 30, 73, 75, 76] Several different parameters and strategies are used for sandblasting, and this could obscure getting the real impact of this procedure on the bond strength. Factors that could affect the bond strength values between the liner materials and denture base resin are the type of lining materials, particle size of sands, blasting pressure and time, test methods, thermocycling, speed of head of testing machine, and thickness of lining material. This review and meta-analysis tried to consider variables in sandblasting including particle size, blasting pressure, and type of liner to identify the effect of this pretreatment in improving bond strength. [11, 15, 25, 31, 33, 36, 78, 84, 85, 87–92].

Quality of resilient lining materials is evaluated by their tensile properties. The bond strength between denture base resin and resilient lining materials is usually assessed by tensile test due to reliable results and also easiness of performance. [35, 85, 93] Results of our meta-analysis showed that in general sandblasting could not improve tensile bond strength significantly. Increasing the bond strength after sandblasting is expected as it provides more bonding surface and creates mechanical locks at bond site, also removing contaminants. [29] It results in irregularities, valleys, depressions, many small pits, and scratches in acrylic resin treated surface. [94, 95] SEM investigation also shows that sandblasted surfaces are rougher and have no debris. [30] Soft lining material could flow into the irregularities of the acrylic resin that resulted in significant effect on adhesive values. [31] However, the size of the irregularities may not be adequate to allow the resilient lining material to penetrate into them without leading to a significant increase in tensile bond strength. [30] As flowing into resin irregularities by the liners is dependent on their viscosity, the liquidity of the elastic materials in a clarified contact angle and surface energy define the penetration. [26, 31] The penetration coefficient (PC) for liquids into a cavity is given by PC = *γ*  cos  *θ*/2*η*, where *γ* is the surface tension, *θ* is the contact angle, and *η* is the viscosity. This can state the lower tensile strengths of sandblasted specimens subjected to the reviewed studies. On the other hand, creation of microcracks, vacancies, and voids during packing the resilient lining material on resin surface may trap air bubbles, compensate for the effect of irregularities for increasing the contact surface, and result in reduced bond strength. [35] The other explanation for strength reduction is the stress induced at the junction of PMMA and soft liner, or stress concentration because of discontinuities on the surface. [46] Another hypothesis for reduced bond strength is separated resin or Al_2_O_3_ particles, which remain in the irregularities of the treated surface and will decrease the bond strength. [32] The rate of Al_2_O_3_ adhesion may be varied in the used denture materials.

The results of meta-analysis of two studies showed that sandblasting do not increase peel bond strength; though shear bond strength increased significantly. Al-Athel et al. designed an investigation of the effect of test methods on bond strengths of the liners. [88] They demonstrated that roughening the surface increased shear bond strength while tensile bond strength decreased. [88] Such finding could be explained by the fact that, in roughened surface, more force is needed to move two surfaces along each other as friction is increased. [88] It should be noticed that the distance between the two surfaces and where the force is applied are the most important factors that could affect shear test values. [13] As debonding begins at the edge of the lining materials, the most similar test to intraoral situations for bonded two-layer dentures is peel test. [7, 60, 96] This test directly measures the debonding force, and the site of applying force is closely similar to the real situation in the mouth. [7, 31, 97] However, it is not possible to catch it at the liner acrylic resin interface directly because the possibility of soft liners tearing is high in peel test. [96] Therefore, thickness of liner seems to be critical as cohesive failure is higher. [89, 98, 99] Moreover, surface energy is different in roughened surface and smoothed one. [100] Pretreatment of denture surface affects its geometry that results in alteration of surface energy. [7, 31] The amount of force recommended in peeling test is related to surface energy of the used materials, so it should be mentioned accurately.

In this study, the reviewed studies were categorized into three groups with strata of small particle size (50 *µ* Al_2_O_3_), medium particle size (60 *µ* Al_2_O_3_ < particle size <125 *µ* Al_2_O_3_), and large particle size (particle size ≥250 *µ* Al_2_O_3_). Studies with small particle size of Al_2_O_3_ (less than 50 *µ*m) showed adverse effect on bond strength of the acrylic resin denture base to resilient material. The result of blasting with particle size in the range of 60 *µ*m to 125 *µ*m showed increasing bond strength; though the difference was not significant. By increasing the particle size to 250 *µ*m, the results again favored not sandblasted groups. The rationale for these findings is that the size of roughening by sandblasting with 50 *µ*m Al_2_O_3_ particles may not be sufficient to allow liner material penetration. [31, 62, 76] As the penetration coefficient of the liners is inversely related to their viscosity, liners with higher viscosity have less penetration into PMMA surface pores. [35] On the other hand, sandblasting with large size particles (250 *µ*m) also reduces the bond strength due to stress concentration of large size particles. Akin et al. suggested sandblasting with particle size of 120 *µ*m in comparison to 50, 60, and 250 *µ*m for maximum bonding. [11].

The second subgroup analysis was performed to investigate the effect of blasting pressure. Pair comparison groups were categorized into two strata based on blasting pressure (0.2 bar ≤ blasting pressure ≤1 bar; 1 bar < blasting pressure ≤ 4 bar). Meta-analysis showed less tensile bond strength for blasted specimens when the blasting pressure was ≤ 1 bar (*p* < 0.001). By increasing the blasting pressure to more than 1 bar, sandblasting became significantly more effective than control group (*p*=0.03). This finding can be explained by more irregularities caused by high pressure of sand steam.

Surface treatment should be selected according to the type of the resilient lining material to achieve acceptable bond strength. [85] Among the included studies, nine evaluated silicone-based soft liners, three used acrylic-based resilient liners, and four evaluated both types of liners. The results showed that groups without any surface treatment had significantly higher tensile bond strength when silicon-based liner was used (*p* < 0.001). Meanwhile, the two studies that used acrylic resin-based liners showed contrary results. Khakbaz et al. showed improved bond strength of acrylic soft liner after sandblasting while Kulkarani et al. indicated higher strength in not blasted group. Overall, there is still controversy about the superiority of silicon and acrylic soft liners. Several articles claimed that the similarity of acrylic resin-based liners to denture bases caused higher bond strength values in comparison with silicon-based lining materials. [75, 78, 83] As methyl methacrylate and ethyl methacrylate are monomers that are basically similar, they can mix through polymerization procedure resulting in a copolymer. Silicone liners do not have any chemical bonding to acrylic denture bases because of their structural differences. [31, 78] On the other hand, some studies demonstrated that heat-polymerized silicone-based resilient lining materials had better bond strength than soft liners that contained plasticizer. These heat-polymerized liners had the greatest bond strengths to acrylic resin denture bases, and the autopolymerized silicone liners had insufficient bonding to acrylic base. [25, 87–92] The most important justification in these articles for superior bond strength of silicon liner in comparison to acrylic-based liner was related to minimal water absorption of silicon-based soft liners. [101].

The high level of heterogeneity in analyses indicates great variation of methodology as well as various influencing factors in the main outcome. These factors include type of liner, size of particle, pressure of blasting, speed of head of testing machine, time of blasting, distance from blasting tip to the specimen, storage condition before testing, and thermocycling. The first three items are discussed in this study with quantitative analyses, and the other five items are presented descriptively. A straight correlation between the tensile strength values and the speed of head of testing machine is reported. [88] It has been shown that the amount of tensile strength between acrylic base and resilient lining material increased significantly up to 40 mm/min speed of machine head, and after that it had reverse effect. [88] Out of our included studies, nine tested the specimens with universal testing machine at a cross head speed of 5 mm/min, and two used cross head speed of 10 mm/min. [9, 11, 33, 75–80] The time of blasting in included studies varied between 10 and 60 seconds, which could be an influencing factor. It has been shown that sandblasting at different distances and angles contributes differences in surface roughness when it is applied to zirconia or titanium materials [102]. However, no study identified the effect of this parameter on the roughness of acrylic resin. Thermocycling also affects the values of bond strength. When resilient liner is immersed in water, it will absorb water and saliva, and the plasticizer and solvent agent will leach out of the liner. The balance between these two mechanisms determines the dimensional stability of the material and bond strength. [16] Two studies evaluated the effect of thermocycling and reported that tensile bond strengths were significantly lower than those in the same sets before thermocycling. [76, 77] Thermocycling could also change the mode of failure to adhesive failure. Nakhaei et al. reported mixed failure for group without thermocycling and adhesive failure for specimen thermocycled between 5 and 55°C for 5,000 cycles. [76].

Taking all of these factors into account, it can be concluded that these factors could affect the final outcome, and more in vitro studies with uniform parameters of testing are encouraged to limit the conflicting factors. The authors could not find any clinical studies that compared the effect of sandblasting on the longevity of bond between denture base and liners, and one of the limitation of this study is that the results are based on in vitro studies. Further clinical studies are needed to indicate the long-term effect of sandblasting as a pretreatment surface preparation.

## 5. Conclusion

Within the limitations of this study, these points can be emphasized:

1. Sandblasting decreases the tensile and peel bond strength of resilient lining materials to denture base resins. However, it improves the shear bond strength.
2. In 50 *µ* Al_2_O_3_ particle size, the amount of bond strength of control group is higher than that of experimental group. However, as the particle size goes beyond 50 *µ*, no significant difference exists between the two groups. In particle size subgroup analysis of shear bond strength, sandblasting with 50 *µ* Al_2_O_3_ resulted in significantly higher shear bond strength. Groups which were sandblasted with 250 *µ* Al_2_O_3_ had no significant difference with no treatment specimen.
3. Meta-analysis showed higher tensile bond strength for control group when the blasting pressure was ≤1 bar. By increasing the blasting pressure beyond 1 bar, sandblasting became significantly more effective than control group.
4. Groups without any surface treatment had significantly higher tensile bond strength when silicon-based liner was used, while the two studies that used acrylic resin-based liners showed no significant difference between control and sandblasting groups.

## Figures and Tables

**Table 1:** Search strategy.

PICO question: what is the effect of sandblasting on bond strength of resilient liners to polymethyl methacrylate (PMMA)?		Items found
Population	1# search ((((((((((((((“tissue conditioner” [Title/Abstract]) OR “soft liner” [Title/Abstract]) OR “lining material” [Title/Abstract]) OR “denture liner” [Title/Abstract]) OR “resilient liner” [Title/Abstract]) OR “denture reline resin” [Title/Abstract]) OR “definitive silicone-based heat-polymerized” [Title/Abstract]) OR “polymethyl methacrylate” [Title/Abstract]) OR “acrylic resin” [Title/Abstract]) OR “denture base” [Title/Abstract]) OR “heat-polymerized polymethyl methacrylate” [Title/Abstract]) OR (“PMMA [Title/Abstract] AND polyamide” [Title/Abstract])) OR “denture bases” [MeSH terms]) OR “polymethyl methacrylate” [MeSH terms]) OR “denture liners” [MeSH terms]) OR “tissue conditioning, dental” [MeSH terms]	16569
Intervention	Search (((((((“silicon carbide paper” [Title/Abstract]) OR “surface pre-treatment” [Title/Abstract]) OR “surface treatment” [Title/Abstract]) OR “pretreated” [Title/Abstract]) OR “silica coating” [Title/Abstract]) OR “sandblasted” [Title/Abstract]) OR “air abrasion” [Title/Abstract]	66164
Outcome	Search (((((((((((“tensile strength” [Title/Abstract]) OR “bond strength” [Title/Abstract]) OR “tensile” [Title/Abstract]) OR “tension bond” [Title/Abstract]) OR “bonding properties” [Title/Abstract]) OR “failure loads” [Title/Abstract]) OR “peel strength” [Title/Abstract]) OR “failure modes” [Title/Abstract]) OR “mode of failure” [Title/Abstract]) OR “failures” [Title/Abstract]) OR “tensile strength” [MeSH terms]) OR “shear strength” [MeSH terms]	107701
PubMed	Search (#1 AND #2) AND #3	212
Scopus	(TITLE-ABS-KEY ((“tissue conditioner”) OR (“soft liner”) OR (“lining material”) OR (“denture liner”) OR (“resilient liner”) OR (“denture reline resin”) OR (“definitive silicone-based heat-polymerized”) OR (“polymethyl methacrylate”) OR (“acrylic resin”) OR (“denture base”) OR (“heat-polymerized polymethyl methacrylate”) OR (“PMMA AND polyamide”) OR (“denture bases”) OR (“polymethyl methacrylate”))) AND (TITLE-ABS-KEY ((“silicon carbide paper”) OR (“surface pre-treatment”) OR (“surface treatment”) OR (“pretreated”) OR (“silica coating”) OR (“sandblasted”) OR (“air abrasion”))) AND (TITLE-ABS-KEY ((“tensile strength”) OR (“bond strength”) OR (“tensile”) OR (“tension bond”) OR (“bonding properties”) OR (“failure loads”) OR (“peel strength”) OR (“failure modes”) OR (“mode of failure”) OR (failures)))	686
Embase	((“Tissue conditioner”) OR (“soft liner”) OR (“lining material”) OR (“denture liner”) OR (“resilient liner”) OR (“denture reline resin”) OR (“definitive silicone-based heat-polymerized”) OR (“polymethyl methacrylate”) OR (“acrylic resin”) OR (“denture base”) OR (“heat-polymerized polymethyl methacrylate”) OR (“PMMA” AND “polyamide”) OR (“denture bases”) OR (“polymethyl methacrylate”)) AND ((“silicon carbide paper”) OR (“surface pre-treatment”) OR (“surface treatment”) OR (“pretreated”) OR (“silica coating”) OR (sandblasted) OR (“air abrasion”)) AND ((“tensile strength”) OR (“bond strength”) OR (“tensile”) OR (“tension bond”) OR (“bonding properties”) OR (“failure loads”) OR (“peel strength”) OR (“failure modes”) OR (“mode of failure”) OR (“failures”))	379
Cochrane	((“Tissue conditioner”) OR (“soft liner”) OR (“lining material”) OR (“denture liner”) OR (“resilient liner”) OR (“denture reline resin”) OR (“definitive silicone-based heat-polymerized”) OR (“polymethyl methacrylate”) OR (“acrylic resin”) OR (“denture base”) OR (“heat-polymerized polymethyl methacrylate”) OR (“PMMA” AND “polyamide”) OR (“denture bases”) OR (“polymethyl methacrylate”)) AND ((“silicon carbide paper”) OR (“surface pre-treatment”) OR (“surface treatment”) OR (“pretreated”) OR (“silica coating”) OR (“sandblasted”) OR (“air abrasion”)) AND ((“tensile strength”) OR (“bond strength”) OR (“tensile”) OR (“tension bond”) OR (“bonding properties”) OR (“failure loads”) OR (“peel strength”) OR (“failure modes”) OR (“mode of failure”) OR (“failures”))	5 trials

**Table 2:** Excluded studies at the full text level with reasons.

Reason for exclusion	Number of excluded articles
Not having sandblasted treatment group	28 [2, 6, 10, 42–66]
Not having any control as untreated group	3 [34, 66, 67]
Not using resilient lining material	3 [41, 68, 69]
Not related to title	1 [70]
Not using PPMA	2 [40, 71]
Not reporting enough data	2 [72, 73]
Not reporting SD	1 [74]

**Table 3:** Characteristics of included studies.

Author, year	Sample size, experimental group-control group	Name of acrylic	Liner material	Size of particle for sandblasting (*µ*)	Pressure (bar)	Time (second)	Distance from tip sandblast to specimen (mm)	Cross head speed (mm/min)	Storage condition before testing	Thermocycling	Mean (SD) of experimental group (MPa)	Mean (SD) of control group (MPa)	Failure mode
Tensile bond strength													
Akin et al., 2011 [9]	15-15	Paladent	Permaflex	50	2	10	10	5	Distilled water at 37°C for 1 week	No	25.25 (2.09)	21.04 (1.29)	NR
Akin et al., 2011 [11]	15-15	Paladent	Permaflex	50	2	10	10	5	Distilled water at 37°C for 1 week	No	0.73 (0.06)	0.88 (0.09)	Control: adhesive, sandblasted: mixed
Akin et al., 2011 [11]	15-15	Paladent	Permaflex	60	2	10	10	5	Distilled water at 37°C for 1 week	No	0.9 (0.21)	0.88 (0.09)	Control: adhesive, sandblasted: mixed
Akin et al., 2011 [11]	15-15	Paladent	Permaflex	120	2	10	10	5	Distilled water at 37°C for 1 week	No	1.2 (0.27)	0.88 (0.09)	Control: adhesive, sandblasted: mixed
Akin et al., 2011 [11]	15-15	Paladent	Permaflex	250	2	10	10	5	Distilled water at 37°C for 1 week	No	1.09 (0.29)	0.88 (0.09)	Control: adhesive, sandblasted: mixed
Khakbaz et al., 2019 [75]	9-9	PMMA	Mollosil soft linear	50	NR	NR	NR	5	Distilled water at 37°C for 1 week	No	3.41 (0.82)	2.02 (0.41)	NR
Khakbaz et al., 2019 [75]	9-9	PMMA	GC Soft Liner	50	NR	NR	NR	5	Distilled water at 37°C for 1 week	No	1.78 (0.36)	0.84 (0.24)	NR
Gorler et al., 2015 [76]	20-20	Meliodent	Molloplast B	125	4	20	10–45 degrees	10	Aqueous incubation at room temperature (24°C) in distilled water	Yes	38.2 (2.3)	43.1 (4.5)	NR
Gorler et al., 2015 [77]	20-20	Meliodent	Molloplast B	125	4	20	10–45 degrees	10	Aqueous incubation at room temperature (24°C) in distilled water	No	41.8 (2.2)	44.4 (5.5)	NR
Gundogdu et al., 2014 [33]	8-8	QC-20	Molloplast B	50	2	10	10	5	Stored in distilled water at 37°C for 1 week	No	1.29 (0.25)	1.32 (0.27)	Adhesive
Gundogdu et al., 2014 [33]	8-8	QC-20	Ufi Gel P	50	2	10	10	5	Stored in distilled water at 37°C for 1 week	No	0.21 (0.03)	0.2 (0.05)	Adhesive
Kulkarni et al., 2011 [78]	10-10	Trevalon	Molloplast B	250	6.2	30	Light contact	5	NR	No	0.998 ± 0.239	1.587 ± 0.207	Sandblasted: adhesive, control: mixed
Kulkarni et al., 2011 [78]	10-10	Trevalon	SUPER-SOFT	250	6.2	30	Light contact	5	NR	No	1.313 ± 0.424	2.622 ± 0.223	Sandblasted: adhesive
Nakhaei et al., 2016 [76]	24-24	Triplex	Molloplast B	110	2	10	10	5	Stored in distilled water at 37°C for 24 hours	No	1.29 ± 0.17	0.9 ± 0.21	Control: mixed, sandblasted: adhesive
Nakhaei et al., 2016 [76]	24-24	Triplex	Molloplast B	110	2	10	10	5	Thermocycled between 5 and 55°C for 5,000 cycles	Yes	1.13 ± 0.14	0.75 ± 0.36	Control: mixed, sandblasted: adhesive
Surapaneni et al., 2013 [79]	10-10	DPI	GC Reline (soft)	250	6.2	NR	NR	5	Kept in incubator at 37°C	No	1.14 ± 0.59	1.16 ± 0.50	Adhesive
Surapaneni et al., 2013 [79]	10-10	DPI	Ufi gel P	250	6.2	NR	NR	5	Kept in incubator at 37°C	No	0.435 ± 0.033	0.480 ± 0.024	Adhesive
Usumez et al., 2004 [30]	10	Paladent	Molloplast B	250	6	60	Light contact	NR	Stored in distilled water at 37°C	No	1.7 ± 0.1	1.1 ± 0.3	Adhesive
Vishwanath et al., 2016 [80]	10–10	Trevalon	Molloplast B (Detax)	50	6.2	30	10	5	NR	No	1.2955 ± 0.0001	1.4757 ± 0.0007	NR
Vishwanath et al., 2016 [80]	10–10	Trevalon	Ufi Gel P (VOCO)	50	6.2	30	10	5	NR	No	0.4431 ± 0.0026	0.6282 ± 0.0025	NR

Shear bond strength													
Hamanaka et al., 2017 [81]	10-10	Acron	Tokuyama Rebase II	50	0.28	10	NR	0.5	Stored in distilled water at 37 8°C for 4 months	Yes	5.6(0.8)	4.6(1.6)	Adhesive
Hawraa Khalid et al., 2017 [82]	10-10	Vertex	Vertex™ Soft	250	4	60	20	1	Conditioned in distilled water at 37°C for 24 hours	No	0.498 (0.02251)	0.569 (0.03071)	NR
Khanna.et al. 2015 [83]	10-10	Trevalon	Luci-Sof	250	0.62	NR	Light contact	20	NR	Yes	18.76 (0.82)	18.27 (0.57)	Adhesive
Khanna.et al. 2015 [83]	10-10	Trevalon	SUPER-SOFT	250	0.62	NR	Light contact	20	NR	Yes	27.42 (1.41)	18.82 (0.57)	Mixed
Takahashi et al., 2001 [29]	8-8	Lucitone 199	GC Kooliner (hard)	50	NR	NR	NR	1	Stored in 37°C distilled water for 1 month	Yes	4.3 (2.5)	2.2 (1.4)	NR
Takahashi et al., 2001 [29]	8-8	Lucitone 199	Triad VLC Reline	50	NR	NR	NR	1	Stored in 37°C distilled water for 1 month	Yes	7.3 (2.5)	2.9 (2.1)	NR
Takahashi et al., 2001 [29]	8-8	Lucitone 199	GC Reline (hard)	50	NR	NR	NR	1	Stored in 37°C distilled water for 1 month	Yes	3.3 (1.4)	2.7 (1.5)	NR

Peel bond strength													
Jacobsen et al., 1997 [31]	20-20	Lucitone 199	BioSoft Better Health Systems	250	0.62	30	NR	50.8	Stored in distilled water	No	43.1 (13.3)	78.5 (24.8)	Cohesive
Jacobsen et al., 1997 [31]	20-20	Lucitone 199	Prolastic	250	0.62	30	NR	50.8	Stored in distilled water	No	16.2 (8.7)	44.7 (22.1)	Cohesive
Korkmaz et al., 2013 [32]	11-11	Paladent	Molloplast B	50	2	30	Light contact	10	In distilled water at 37°C for one week	No	3.16 (0.64)	3.64 (0.49)	Control: mixed, test: adhesive
Korkmaz et al., 2013 [32]	11-11	Rodex	Molloplast B	50	2	30	Light contact	10	In distilled water at 37°C for one week	No	4.46 (0.26)	3.89 (0.48)	Control: mixed, test: adhesive
Korkmaz et al., 2013 [32]	11-11	Deflex	Molloplast B	50	2	30	Light contact	10	In distilled water at 37°C for one week	No	4.58 (0.54)	3.1 (0.55)	Control: mixed, test: adhesive

**Table 4:** Risk of bias assessment.

Author, year	Specimen randomization	Single operator protocol implementation	Blinding of the testing machine operator	Presence of a control group	Standardization of sample preparation	Failure mode evaluation	Following the manufacturer's instructions	Description of sample size calculation	Discarded unacceptable samples	Risk of bias
Akin et al., 2011 [9]	Yes	NM	NM	Yes	Yes	No	Yes	NM	Yes	Medium
Akin et al., 2011 [11]	Yes	NM	NM	Yes	Yes	Yes	Yes	NM	Yes	Low
Khakbaz et al., 2019 [75]	Yes	NM	NM	Yes	Yes	No	Yes	NM	Yes	Medium
Gorler et al., 2015 [77]	Yes	NM	NM	Yes	Yes	No	Yes	Yes	Yes	Low
Gundogdu et al., 2014 [33]	Yes	NM	NM	Yes	Yes	Yes	Yes	Yes	Yes	Low
Kulkarni et al., 2011 [78]	Yes	NM	NM	Yes	Yes	Yes	Yes	NM	Yes	Low
Nakhaei et al., 2016 [76]	Yes	NM	NM	Yes	Yes	No	Yes	NM	Yes	Medium
Surapaneni et al., 2013 [79]	Yes	NM	NM	Yes	Yes	Yes	Yes	No	Yes	Low
Usumez et al., 2004 [30]	Yes	Yes	No	Yes	Yes	Yes	Yes	No	Yes	Low
Vishwanath et al., 2016 [80]	Yes	NM	NM	Yes	Yes	No	Yes	Yes	NM	Medium
Hamanaka et al., 2017 [81]	Yes	NM	NM	Yes	Yes	Yes	Yes	NM	Yes	Low
Hawraa Khalid et al., 2017 [82]	Yes	NM	NM	Yes	Yes	No	Yes	NM	Yes	Medium
Khanna et al. 2015 [83]	Yes	NM	NM	Yes	Yes	Yes	Yes	NM	Yes	Low
Takahashi et al., 2001 [29]	Yes	NM	NM	Yes	Yes	No	Yes	NM	Yes	Medium
Jacobsen et al., 1997 [31]	Yes	NM	NM	Yes	Yes	Yes	Yes	NM	Yes	Low
Korkmaz et al., 2013 [32]	Yes	NM	NM	Yes	Yes	Yes	Yes	NM	Yes	Low

NM: not mentioned.

## Data Availability

The data that support the findings of this study are available from the corresponding author upon reasonable request.
